# The Roles of Mesenchymal Stromal/Stem Cells in Tumor Microenvironment Associated with Inflammation

**DOI:** 10.1155/2016/7314016

**Published:** 2016-08-18

**Authors:** Drenka Trivanović, Jelena Krstić, Ivana Okić Djordjević, Slavko Mojsilović, Juan Francisco Santibanez, Diana Bugarski, Aleksandra Jauković

**Affiliations:** ^1^Laboratory for Experimental Hematology and Stem Cells, Institute for Medical Research, University of Belgrade, Dr. Subotića 4, P.O. Box 102, 11129 Belgrade, Serbia; ^2^Laboratorio de Bionanotecnologia, Universidad Bernardo O Higgins, General Gana 1780, 8370854 Santiago, Chile

## Abstract

State of tumor microenvironment (TME) is closely linked to regulation of tumor growth and progression affecting the final outcome, refractoriness, and relapse of disease. Interactions of tumor, immune, and mesenchymal stromal/stem cells (MSCs) have been recognized as crucial for understanding tumorigenesis. Due to their outstanding features, stem cell-like properties, capacity to regulate immune response, and dynamic functional phenotype dependent on microenvironmental stimuli, MSCs have been perceived as important players in TME. Signals provided by tumor-associated chronic inflammation educate MSCs to alter their phenotype and immunomodulatory potential in favor of tumor-biased state of MSCs. Adjustment of phenotype to TME and acquisition of tumor-promoting ability by MSCs help tumor cells in maintenance of permissive TME and suppression of antitumor immune response. Potential utilization of MSCs in treatment of tumor is based on their inherent ability to home tumor tissue that makes them suitable delivery vehicles for immune-stimulating factors and vectors for targeted antitumor therapy. Here, we review data regarding intrusive effects of inflammatory TME on MSCs capacity to affect tumor development through modification of their phenotype and interactions with immune system.

## 1. Introduction

Establishment of the tumor microenvironment (TME) is the most important condition for the sustention of tumor growth. Firstly, formation of TME has been characterized in primary tumors, while today it is clear that creation of tumor-supporting niches at distant sites of the body is required for metastasis progression [1]. Namely, TME plays crucial role in each step of tumor development: oncogenic transformation/initiation, angiogenesis, immune surveillance escape, metastasis, survival of circulating tumor cells in blood, tumor cell stemness, and resistance to radio- and chemotherapy. In order to foster tumor propagation at distant niches, tumor cell-derived factors act in systemic manner, not only locally, thus providing spreading and recurrence of disease [2]. Moreover, supporting a dormancy of tumor cells, TME can provide survival of clinically inconspicuous and hardly detectable metastasis in the body for long time, which is implicated in appearance of relapse. Besides tumor cells, TME includes endothelial cells, fibroblasts, mesenchymal stromal/stem cells (MSCs), and various immune cells, which are together with cytokines and growth factors embedded in tumor stroma endowed with specific physical (oxygen pressure) and biomechanical cues [3]. Thus, understanding of the multiplex visage of TME composition is important not only in investigation of molecular basis of cancer disease, but also in bioengineering of tumor tissue for investigation of disease development, as well as for drug efficacy and safety testing [4]. Reciprocal communication between cells and their microenvironment is important not only for normal tissue development and homeostasis, but also for tumor growth and progression [5]. After many years of investigations, multifaceted roles of TME are today understood as consequence of its active and dynamic composition. It could be speculated that the active nature of TME is actually reflection of the dynamic phenotype or plasticity of cellular compartments within [6]. Heterogeneity of tumor cell population in tumor tissue is well known, but now it is clear that such heterogeneity may be mastered and modified by nontumor cells in TME. This interplay between tumor and nontumor cells in TME is bidirectional. Obligatory condition for tumor development is evasion of antitumor immune response. Tumor cells have various different instruments to avoid destruction by immune system and importantly to control the balance of inflammation in TME and behavior of immune cells. As third player, MSCs have received great attention in cancer research due to their outstanding properties, tumor-homing ability, dynamic phenotype, and immunoregulatory activity [7]. It looks like the evolution of tumor includes adjustment of complete TME. Importantly, TME shapes phenotypes and functions of MSCs and immune cells assigning them tumor-supportive roles. Recently, it has been proposed that epigenetic modifications (histone modifications, changes in expression of DNA methyltransferases, and factors of chromatin modification and microRNA) in tumor as well as in stromal compartment of TME can appear during reprogramming process and contribute to the tumor progression [8–10].

## 2. Persistence of Chronic Inflammation and Hypoxia in TME

Healing of normal damaged tissue includes inflammatory phase which precedes proliferation of resident epithelial and mesenchymal cells and tissue remodeling. Inflammatory phase is limited up to 14 days and include recruitment and infiltration of neutrophils, macrophages, and lymphocytes which play crucial role in secretion of inflammatory cytokines, growth factors, and chemokines involved in resident progenitor cells activation and tissue regeneration and repair [11]. The inflammatory response is finalized by elimination of harmful agents and repair of tissue damage through the differentiation of resident or recruited stem/progenitor cells or by forming scar made of connective tissue to save tissue integrity [12].

Contrary to normal tissue, when prolonged inflammation occurred, “wounds that do not heal” or “overhealing wounds” [13, 14] can cause development of tumor tissue [2]. Wound healing in normal and tumor tissue refers to the engagement of growth and differentiation processes in epithelial as well as stromal components of tissue. In contrary to normal tissue homeostasis and repair, development of neoplastic tissue is characterized by loss of control of many cellular, molecular, and biochemical processes [9].

Chronic inflammation is involved in pathogenesis in many human diseases, while in some circumstances it can contribute to oncogenesis as well [15]. State of chronic inflammation and infiltration of immune cells in TME of solid tumors had been proposed in 19th century by German pathologist Rudolf Virchow [16] and today it is known that tissue injury and chronic inflammation are important risk factors for tumor development. Besides deranged apoptosis, response to growth signals, necessity for growth factors, angiogenesis, replicative potential, and invasion of tumor cells, inflammation is assigned as the seventh hallmark of cancer [17, 18]. Therefore, it is very important to identify cellular and humoral factors which are responsible for resolution of inflammation in normal tissue repair process as well as in development of malignant transformation [19].

One of the proposed contributors and a possible causer of inflammation in tumor is low oxygen (O_2_) concentration (hypoxia). It is known that initial (over)growth of transformed cells is followed by insufficient angiogenesis, which results in hypoxia (partial pressure of O_2_ 1.1–1.3%) in the middle of tumor tissue. In this region, many tumor cells undergo necrosis, while the peripheral regions survive and adapt to hypoxia thanks to the hypoxia-inducible factor 1 (HIF-1) dependent gene expression, where transformed cells become more glycolytic [12, 20]. In general, swelling tissue forces cells to consume oxygen to survive hard conditions in inflamed regions, thus creating their hypoxic nature. It looks that cancer stem cells are more adapted to hypoxic conditions than differentiated tumor cells, due to their quiescence and low-energetic demands [21]. However, because tumor cells are not engaged in tissue homeostasis, tumor-associated hypoxia is a pathophysiologic state [22–24], caused by corrupted microcirculation which became a central issue and impediment in tumor biology and treatment, since hypoxic regions show resistance to the radio- and chemotherapy thus being negative prognostic and predictive indicators [25, 26]. Moreover, it has been demonstrated that the hypoxia and HIF-1 stabilization may lead to reduced activity of macrophages, which become unable to phagocyte tumor cells. Also, the HIF-1 stimulates production of CD47 in tumor cells, a cell surface protein that enables tumor cells to avoid destruction by macrophages [27].

Activities of hypoxia and inflammation in tumor are intertwined at the molecular and cellular level [28, 29]. Importantly, reacting to hypoxia and HIF-1 activation, tumor and normal stromal cells can express alarmin receptors which bind alarmins released by necrotic cells in tumor tissue. Signaling triggered by alarmin receptor leads to the activation of nuclear factor kappa-light-chain-enhancer of activated B cells (NF-*κ*B) and expression of proinflammatory genes, thus contributing to tumor progression. NF-*κ*B activation by HIF-1 triggered by hypoxia or accumulated cytosolic reactive oxygen species lead to production of inducible proinflammatory enzymes: inducible 5-lipoxygenase (5-LOX), NADPH-oxidases (NOX), inducible nitric oxide synthase (iNOS), inducible cyclooxygenase (COX2), and inducible heme-oxygenase-1 (HO-1) [12]. Considering that activation of the NF-*κ*B by hypoxia and increment of various cytokines production has been observed in bone marrow MSCs [30], it is plausible to speculate that tumor-associated hypoxia can stimulate production of tumor-supporting factors in MSCs in NF-*κ*B-dependent manner.

On the other hand, unavoidable hypoxia in TME can be fertile ground for generation of regulatory immune cells, such as regulatory T cells (Treg) and myeloid-derived suppressor cells (MDSCs) in tumor [31]. Importantly, HIF-1 is pertinent factor involved in regulation of metabolic commitment of immune cells, immunometabolism, thus controlling their activation, proliferation, and effector functions [32]. Complexity of metabolic configuration of immune cells is related to their energy demands, synthesis of biomolecules, and subsistence [33]. However, it has been demonstrated that HIF-1 controls generation of tolerogenic immune cells in TME, such as Treg [34], MDSCs [35], and tumor-associated macrophages (TAMs) [36]. These evidences indicated that hypoxia could be regulator of inflammation in TME, by controlling metabolic state of immune cells. Although hypoxia is known as regulator of stem cell metabolism [37], there is scant data about influence of hypoxia on immune status and activities of MSCs and this issue should be further explored because it may be expected that hypoxia governs immunosuppressive properties of MSCs. These answers can help to better understand immune properties of MSCs [38] and their participation in pathogenesis of cancer.

## 3. MSCs: Recruitment and Functions in TME

Mesenchymal stromal/stem cells (MSCs) are widely distributed in TME, where they communicate with other cells and participate in the tumor development. Moreover, besides myofibroblasts, pericytes, and hematopoietic cell-derived fibrocytes, MSCs are also identified as possible cell origin of tumor-associated fibroblasts (TAFs). Because of heterogeneity within cells in the TAF population, their unresolved origin, many intermediate populations occurring during their context-specific lineage commitment, and cellular plasticity [39–41], there is still no consensus in the literature regarding exactly defined phenotype and function of TAFs in TME. In this part, we will discuss MSCs potential to respond to various TME-derived stimuli, adapting their recruitment to the tumor sites, phenotype, and functional properties.

As mentioned above, MSCs contribute to maintenance of normal tissue homeostasis, while today it is clear that these cells can influence tumor development. Namely, MSCs, which have been recently assigned as “medicinal signaling cells” [41] or “ambulatory cells” [42], have capacity to home sites of inflammation, manifest immunosuppressive properties, and differentiate into various cell types. For the same reasons, MSCs have received great attention in cancer research. It has been proposed that normal MSCs (N-MSCs) and tumor MSCs (T-MSCs), although sharing similar phenotype properties, could have different influence on tumor development, demonstrating supportive role of T-MSCs in tumor growth [43, 44]. Aforementioned data indicated conspicuous importance of TME not only in tumor development, but also in cancer recurrence and poor prognosis of patients. Moreover, it has been shown that expression of mesenchymal genes in tumor cells and mesenchymal signature in cancer transcriptome are related to aggressiveness of cancer [45]. Also, molecular profiling of stromal cells from various human tumors can provide significant diagnostic information [46].

It has been accepted that MSCs participate in each step of tumor development: evasion of immune surveillance, promotion of tumor angiogenesis, resistance to chemotherapeutics, invasion and metastasis, and induction of stem-like properties in tumor cells [43].

MSCs are implicated in tumor cells maintenance and immunosurveillance evasion creating selective pressure for tumor cells which escape antitumor immune response and conditioning tumor progression [47]. This selective pressure has been also found to be key factor for acquisition of neoantigens by tumor cell. Evasion of immune attack by tumor cells is caused by their abilities to edit neoantigens unrecognizable by adaptive immune system, acquire the resistance to apoptotic/necrotic mechanisms, and suppress the adaptive and innate immune response [39, 40, 48].

However, as context-dependent activation of stromal cells has been demonstrated in healing of normal tissue, recruitment and activity of stromal cells in tumors look to be subordinated by TME circumstances. It is likely that tumor-associated inflammation, whether it precedes or follows tumor development, is part of the normal response to injury and infection that has been converted by tumor cells to their advantage [16].

Capacity of MSCs to regulate immune response is major advantage for their use in cell-based therapies and tissue engineering. However, this feature is not constitutive property of MSCs, and it is important to note that microenvironment stimuli can shape and modify immunosuppressive potential of MSCs. Therefore, various factors can trigger different immune status and activity of MSCs, leading to stimulation or suppression of immune response [49–51]. However, mechanisms involved in the regulation of immunological properties of MSCs are still not clearly understood.

**Figure 1:**
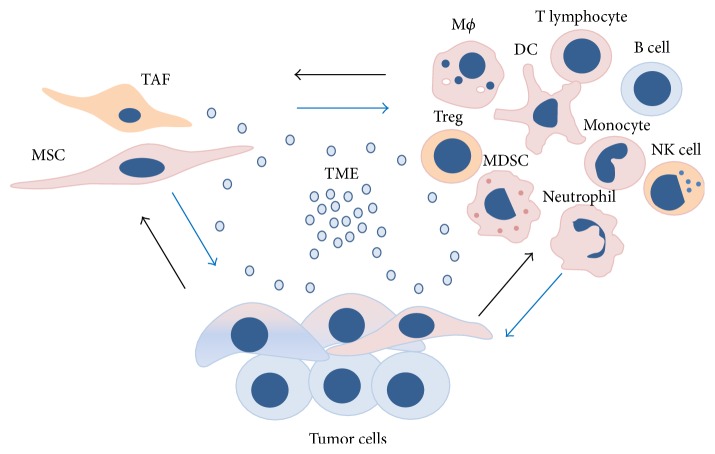
Tumor microenvironment (TME) as a fertile ground for dynamic cell phenotype and function. Composition of TME implies immediate vicinity of mesenchymal stromal/stem cells (MSCs), tumor-associated fibroblasts (TAFs); immune cells: macrophages (MФ), regulatory T cells (Treg), myeloid-derived suppressor cells (MDSCs), natural killer (NK) cells, dendritic cells (DCs), monocytes, neutrophils, T lymphocytes, B cells, and heterogenic population of tumor cells. Reciprocal communication within cellular compartment is accomplished through the paracrine network.

Due to abundance of various factors and infiltrated immune cells in TME, it is justifiable to speculate on significance of interactions between MSCs and immune cells in the context of tumor development. In case of solid tumors, it has been described that heterogenic population of neoplastic cells collaborates with MSCs and immune cells, forming a “vicious triangle” [46] or maybe “vicious circle” of tumor development (Figure 1).

On the basis on previous data, where the C-X-C chemokine receptor type 4 (CXCR4)/stromal-derived factor-1 (SDF-1) (or CXCL12) has been shown as major axis implicated in regulation of hematopoietic stem cells recruitment and retention in bone marrow stroma, it has been proposed that the same axis is involved in regulation of MSC recruitment to tumors [52, 53]. Also, it has been demonstrated that tumor cells which express CXCR4 home bone marrow and that SDF-1 attracts MSCs from bone marrow or other sites in body to tumor tissue [54]. Moreover, it has been reported that soluble factors of tumor cells can induce secretion of SDF-1 by MSCs and enhance their motility [55]. However, precise role of SDF-1 in ability of MSCs to home tumor tissue is not completely elucidated, as some studies have showed that tumor cells produce no SDF-1. Moreover it has been proposed that macrophage migration inhibitory factor (MIF) is major regulator of MSC migration toward tumors. There has been observed physical interaction between MIF and CXCR2, CXCR4, and CD74, although only CXCR4 is dominant receptor recognized by MIF in context of tumor homing. Also, it has been shown that activation of the Mitogen-Activated Protein Kinases (MAPK) pathway in MSCs has been necessary for tumor homing [56].

As final step of the diapedesis, migration of bone marrow MSC toward tumor has been described to be dependent on matrix metalloproteinases (MMPs) expression in MSCs [53]. It has been observed that MSC tropism toward glioma cells is regulated by MMP-1-activated CXCR4/SDF-1 axis [57]. Also, tropism of adipose tissue MSCs toward brain tumors has been demonstrated by Feng et al., 2014 [58]. Interestingly, in this study, it has been reported that hypoxia increases motility and tropism of MSCs toward glioblastoma* in vitro* and* in vivo*, without change in their differentiation toward TAFs.

It could be speculated that retention of MSCs in the TEM of breast cancer is also regulated by the presence of hypoxia in various tumor zones. It has been shown that hypoxia induced HIF-1 stabilization mediates bidirectional paracrine signaling between breast cancer cells and MSCs which stimulates breast cancer metastasis [59].

On the other hand, MSC niche can be target for disseminated tumor cells. The human bone marrow can become populated with tumor cells of breast cancer patients [60], as well as prostate cancer [61]. It has been demonstrated that disseminated tumor cells inhabiting bone marrow stroma can lead to reduction of hematopoiesis and level of SDF-1. Moreover, it has been found that disseminated tumor cells express markers of hematopoietic cells presented in bone marrow, thus mimicking microenvironmental marker pattern expression, which complicate detection of tumor cells and may contribute to inefficiency of therapeutics [61]. Also, CXCR4/SDF-1 signaling has been shown to be involved in the homing and proliferation of leukemia cells in acute myeloid leukemia (AML), chronic myeloid leukemia (CML), and other hematologic malignancies. It is important to note that CD44 and very late antigen-4 (VLA-4, *α*4*β*1-integrin) receptor expressed by leukemia cells govern their adhesion to bone marrow stromal cells in the niche and consequent induction of antiapoptotic effects that support leukemia cell survival. Furthermore, bone marrow MSCs have been shown to up-regulate the secretion of several inflammatory factors (interleukin- (IL-) 6, vascular endothelial growth factor (VEGF), SDF-1, and tumor necrosis factor-*α* (TNF-*α*)) as a result of their direct interaction with myeloma cells, which in turn promote supportive role of TME in undisturbed survival, growth, and development of multiple myeloma [62].

Moreover, it has been demonstrated that CD138-myeloma cells, which have elevated expression of SDF-1, induce higher stiffness in MSCs from multiple myeloma patients than in normal MSCs through the induction of SDF-1/CXCR4/AKT signaling [55].

In addition, the link between obesity and tumor formation has been accepted, where tumor-supporting role of inflammation, adipokines, and chemokines has been suggested. It has been found that adipose tissue MSCs contribute to tumor-promoting state of TME, through increased secretion SDF-1, VEGF, chemokine ligand 5 (CCL5), platelet-derived growth factor D (PDGF-D), and TGF-*β* in response to presence of tumor cells. Moreover, hypoxia localized in adipose tissue, has been shown to be associated with elevated levels of proinflammatory cytokines, TNF-*α*, and IL-6, which are known as important factors in tumorigenesis [63].

## 4. The Issue of Tumor-Associated Fibroblast (TAF)

In the literature, the term of TAFs is commonly used for cell population actively participating in TME constitution and cancer progression, and despite their poor defined identity, it covers a heterogeneous population of stromal cells displaying various phenotypes. Although fibroblasts are most frequently recognized resident cell type in tumor stroma, they possess very dynamic context-dependent phenotype and functions. Recently, three states of fibroblasts in context of TME have been proposed: resident fibroblasts, tumor permissive or suppressive “primed” fibroblasts, and tumor-promoting “activated” fibroblasts [64]. However, it has been suggested that TAFs can originate from recruited or resident fibroblasts, stem/progenitor, or immune cells deriving from surrounding tissue or bone marrow. Also, TAFs can ensue from tumor cells in the process of the epithelial-to-mesenchymal transition (EMT). Within stem/progenitors compartment, MSCs are proposed as one of potential candidates for origin of TAFs.

Namely, MSCs can acquire myofibroblastic phenotype of TAFs [46, 65]. After activation and differentiation into myofibroblasts, TAFs produce several mesenchymal proteins such as fibroblast-specific protein (FSP-1), fibroblast-activating protein (FAP), vimentin, and *α*-smooth muscle actin (*α*-SMA). As expression of *α*-SMA, FSP1, FAP, chondroitin sulfate proteoglycan (NG2), and PDGFR-*β* is also common for normal fibroblasts, it is proposed that bulk population of TAFs consist of two subpopulations: fibroblasts and myofibroblasts [66]. Also, it has been described that bone marrow derived cells differentiate into TAFs which secrete large amount of extracellular matrix (ECM) components, but also mediators of tumor cells invasion such as metalloproteinase-13 [67].

During normal tissue repair, activated fibroblasts migrate toward inflamed region, proliferate, and differentiate into highly contractile myofibroblasts. Presence of various growth factors, such as TGF-*β*, but also mechanical properties of extracellular matrix can master differentiation of myofibroblasts [9].

In tissue repair process, transition of fibroblast into myofibroblast is the most important physiological event, while its disruption leads to fibrosis and eventually to tumorigenesis. Several studies have demonstrated that differentiation of stem/progenitor cells into myofibroblasts is regulated by TGF-*β*. It has been demonstrated that TGF-*β* regulate initial as well as later phase of myofibroblast differentiation of adipose tissue MSCs towards stationary myofibroblasts [68]. Differentiation of adipose tissue MSCs into TAFs, induced by breast tumor cells-derived TGF-*β*, is regulated through Smad3 signaling [65]. Moreover, it has been demonstrated that adipose tissue-derived MSCs develop contractile myofibroblastic phenotype after treatment with TGF-*β*, increasing expression of *α*-SMA and ECM proteins, such as collagen and fibronectin. Interestingly, in this study, it has been described that other tissue factors, such as fibroblast growth factor (FGF), induce redifferentiation of myofibroblasts into fibroblast-like cells [69].

Also, it has been demonstrated that exosomes secreted by prostate cancer cells induce subsequent phenotype changes of bone marrow MSCs, inhibiting their adipogenic and promoting myogenic differentiation through stimulation of *α*-SMA expression. In this study, the role of TGF-*β* in exosome-mediated effects on MSC differentiation has been confirmed, while MSC-derived myofibroblasts have been shown to possess tumor-promoting activity [70].

However, nondistinctive origin of TAFs indicates that TAFs may be defined as dynamic and transiently state of fibroblast-like cells, rather than certain phenotype. TAFs can be retained in TME for long time and it may be speculated that TME, with all its unsteady elements, contributes to maintenance of TAF state [71]. Moreover, it has been proposed that maintenance of TAF state in TME could be ensured through epigenetic alteration in tumor-associated stromal cells. It has been observed that tumor-associated stromal cells promote epithelial tumor cells growth, by activated Wnt/*β*-catenin signaling. Also, overexpression of chromatin remodeling protein Hmga2, an epigenetic regulator in the stroma, induces tumorigenic lesions in the neighboring epithelium in a Wnt-dependent manner [72].

According to these data, it is clear that TME may contribute to reorganization of lineage commitment of various cells, especially of MSCs through modulation of their differentiation, indirect/direct transdifferentiation, and/or cell fusion of MSCs with tumor cells [73]. Therefore it can be speculated that TAF may present example of tumor-biased and redirected phenotype of MSCs.

## 5. TAFs Ensure Tumor Enhancing Inflammation and Immunosuppression

There are many evidences of the proinflammatory role of TAFs. It has been observed that TAF from human colorectal liver metastasis in response to TNF-*α* and consequent activation of NF-*κ*B produce elevated level of proinflammatory cytokine IL-8 [74]. Additionally, microarray results demonstrated high expression of a number of factors such as CXCL2, IL-6, IL-1*β*, CXCL1, CXCL5, COX-2, MMP12, MMP3, and osteopontin (OPN) in TAFs founded in skin, mammary, and pancreatic tumors. This inflammatory signature of TAFs promoted macrophage recruitment, neovascularization, and tumor growth [75, 76]. Additionally it has been observed that binding of IL-1*α* produced by pancreatic tumor cells to IL-1 receptor 1 on TAFs strongly induce production of inflammatory factors by TAFs, contributing to persistence of inflammatory TME [75]. During early tumorigenesis, TAFs have been shown to produce many proinflammatory mediators which can be further stimulated by factors produced by resident immune cells. Also, TAFs have been observed to secrete CCL2 and recruit macrophages in TME. On the other side, TGF-*β*-producing TAFs inhibit natural killer (NK) cell and cytotoxic T lymphocytes (CTL) activation, while stimulating generation of Treg [16].

Additionally, in comparison to normal fibroblasts, TAFs have been shown to express coregulatory molecules, two ligands of programmed death (PD) receptor, PD-L1 and PD-L2, and strongly suppressed proliferation of T lymphocytes. These results indicated that TAFs collaborate with tumor cells and TME in order to establish immunosuppressive milieu and facilitate tumor evasion from immune system [77, 78].

Therefore, it is justifiable to view TAFs as example of “extended phenotype” that was conceived by Dawkins in 1982 [79]. TAFs can serve as an example of how TME educate nontransformed and genetically stable stromal cells to support tumor growth [71, 80]. In similar manner, TME can educate MSCs. TAF represent polarized cell type, which in dependence of type of microenvironmental cues can acquire phenotype I (antitumorigenic) or phenotype II (protumorigenic). TNF-*α* and lipopolysaccharide (LPS) have been proposed to be inducers of phenotype I, while phenotype II could be induced by CXCL14, hedgehog (Hh), or TGF-*β* [66]. Also, similar polarization has been suggested for MSC population [51, 81]. Complexity of determination of TAF as individual phenotype has been nicely described by Xiong et al., 2015 [82], where the author has proposed that TAFs could be generated at various points in intertwined cascades of mesenchymal and hematopoietic stem cells differentiation [83–85].

## 6. MSCs as Regulators of Inflammation

Evidences about potential of MSCs to suppress immune response due to direct or paracrine communication with immune cells are described elsewhere [49]. After establishing their immunomodulatory potential* in vitro *and* in vivo*, bone marrow MSCs have been introduced into the clinical settings, in which these cells showed their therapeutic effects in treatment of acute grade IV GvHD after bone marrow transplantation [86]. However, as in case of immune cells, immunosuppressive potential of MSCs is context-dependent [38]. As mentioned above, the existence of two functional phenotypes of MSCs, M1 (proinflammatory) and M2 (anti-inflammatory), has been proposed [81, 87], which is similar as in population of macrophages, thus implying the possibility of immune cell-like behavior of MSCs [38]. As sensors of inflammation, MSCs respond to various microenvironment stimuli, by changing or adjusting their secretory repertoire and immune activity. Therefore, presence of various cytokines in inflammatory TME, such as TNF-*α*, interferon (IFN)-*γ*, IL-6, IL-1, and TGF-*β*, can govern immune activities of MSCs [43]. Also, it has been described that MSCs can alter activation of NF-*κ*B in macrophages thus controlling their polarization toward M1 or M2 phenotype [87]. Similarly, it has been found that lung tumor cell factors can induce proinflammatory phenotype in MSCs by activation of NF-*κ*B [88], which is known to induce production of inflammatory factors with tumor-supporting roles such as IL-6, TGF-*β*, and IL-1 [89]. Thus, it seems that MSCs can behave like immune cells, responding to inflammatory factors of TME and modifying their secretory and activity profile to more immunosuppressive.

## 7. IL-6

Contribution of MSCs to tumor development is highly dependent on IL-6 activities. It is well known that IL-6 in joint action with hypoxia supports immune evasion of tumor cells, polarizing macrophages to suppressive M2 phenotype [90] and promoting Th17 immune response [91]. It has been found that senescent umbilical cord MSCs produce high amount of IL-6, which is shown to be major mediator of MSCs and tumor cells crosstalk [92]. IL-6 secreted from MSCs activates STAT3 phosphorylation in breast tumor cells, stimulating their proliferation and migration [93]. Moreover, it has been demonstrated that tumor cells and especially their secreted molecules or extracellular vesicles induce higher expression of IL-6 in MSCs, thus educating them to acquire tumor-supporting properties [94]. Also, it has been shown that CD90^+^ cells in TME, assigned as MSC-derived TAFs, are major source of IL-6 in colorectal cancers. Besides, IL-6 has been shown to induce expansion of cancer stem-like cells in tumors, as well as Th17 immune response, thus stimulating tumor progression [95]. As has been shown that expression of IL-6 by MSCs is dependent on their differentiation stage, it could be speculated that TME can regulate expression and production of IL-6 in MSCs affecting their stem-like features. It has been proposed that IL-6 produced by TAFs leads to inhibition of monocyte and macrophage differentiation and function, contributing to final immunosuppression and creation of tumor-promoting microenvironment [96]. Additionally, it has been reported that presence of IL-6, as well as TNF-*α* and IL-1, can lead to neoplastic transformation, through activation of the NF*κ*B and increment of cyclin D1 in normal cells, thus connecting tumorigenesis with inflammation [97]. As MSCs can be source of these proinflammatory cytokines, it is plausible to speculate about contribution of MSCs to neoplastic phenotype development. Although IL-6 has been well described as important factor in regulation of MSCs and tumor cells properties, its mechanisms of action in crosstalk of MSCs and tumor cells are still not completely understood.

## 8. TGF-*β*

As has been recognized as an important player during the multistep cascade of tumor development and progression, TGF-*β* is one of the most investigated immunosuppressive cytokines in TME. However, dual role of TGF-*β* in tumor development is well known. Namely, in early stages of tumor development, TGF-*β* has antiproliferative effect on tumor cells, inducing cell cycle arrest. In contrary, in later stages, TGF-*β* has tumor-promoting role, activating transcriptional factors Smad3/4 and Wnt, supporting EMT and stemness of tumor cells [97]. As mentioned above, TGF-*β* plays critical role in crosstalk of cells within TME, especially differentiation/transdifferentiation of MSCs or fibroblasts into activated tumor-promoting TAFs [64]. Additionally, it has been described that TGF-*β* and hypoxia present in tumor tissue can synergistic master properties of TAFs and TAMs, favoring tumor growth [98]. It has been demonstrated that gastric tumor cells force differentiation of MSCs toward TAFs, through activation of TGF-*β*/Smad2 pathway in MSCs [99]. Moreover, it has been shown that ovarian tumor cells can decrease activity of NK cells via TGF-*β*, thus contributing to evasion of antitumor immunity [100]. TGF-*β* is also known to be responsible for inhibition of T lymphocytes proliferation, disabling immune system to destroy tumor [101]. Interestingly, it has been found that MSCs produce TGF-*β*, which can act in an autocrine manner, by activating Smad3 pathway and reducing production of iNOS in MSCs, thus contributing to immunogenicity of MSCs [102]. Expression of TGF-*β* in adipose tissue MSCs can be stimulated by inflammatory cytokines presented in TME, such as IFN-*γ* and TNF-*α*. It has been reported that TGF-*β* produced by cytokine-primed adipose tissue MSCs contribute to EMT, migration, and invasiveness of breast cancer cells [103]. These findings indicate important role of TGF-*β* not only in regulation of tumor cells behavior, but also in determination of MSC activity in inflammatory TME.

## 9. IL-1

IL-1*α* and IL-1*β* can be produced by tumor as well as stromal cells. These cytokines are involved in development of sterile inflammation in some tumors, such as melanomas. It has been demonstrated that IL-1*α*/*β* produced by tumor cells can augment capacity of MSCs-derived TAFs to suppress proliferation and function of T lymphocytes. Also it has been shown that these effects are achieved through stimulation of COX-2 and immune-checkpoint inhibitors PD-L1 and PD-L2 expression in MSCs-derived TAFs [104]. Moreover, it has been demonstrated that* in vitro* transformation of MSCs contributes to their immunosuppressive capacity, by causing reduction of their immunogenicity and enhancing capacity to suppress proliferation of T lymphocytes. Despite disrupted IFN-*γ* signalization, activity of IL-1*β* sustains immunosuppressive potential of transformed MSCs. Namely,* in vitro* transformed MSCs possess higher expression of IL-1*β*, which acts as intrinsic mediator of inflammation and masters relation of tumorigenicity and immune surveillance escape in an autocrine manner [105]. Additionally, increment of IL-1*β* in TME can lead to stabilization of HIF-1 in tumor cells and contribute to tumorigenesis through activation of genes implicated in metabolism, angiogenesis, and migration [98]. It has been demonstrated that IL-1*α* enhances production of TGF-*β* in MSCs, thus contributing to immunosuppressive functions of MSCs and promotion of prostate cancer cell immune surveillance evasion [106].

## 10. Interaction of MSCs with Immune Cells in TME

### 10.1. Innate Immune Cells

Interacting with compartments of innate immunity in TME, MSCs are able to manifest both anti-inflammatory and proinflammatory properties. However, these features and activities of MSCs are of implausible importance for state of TME, antitumor immunity, and tumor development.

Macrophages and neutrophils are the most abundant population of myeloid immune cells in TME. Due to well-known plastic nature and ability to adapt phenotype in different environmental conditions, macrophages play important role in normal tissue homeostasis, as well as tumorigenesis. Depending on external signals presented within TME, macrophages reversibly change their profile. Importantly, within heterogenic population of macrophages in TEM, TAMs have been characterized according to their functional properties and ability to enhance tumor growth, survival, angiogenesis, and progression [107]. High levels of IL-4, GM-CSF, and TGF*β* present in TME provide macrophages to undergo transition from proinflammatory type M1 to anti-inflammatory M2 phenotype [16]. Similar to macrophages, MSCs are involved in steady-state tissue homeostasis and tumorigenesis and therefore interactions of these cells have received great attention today. It has been observed that in response to factors produce by macrophages type M1, MSCs acquire immunosuppressive phenotype and produce higher levels of iNOS, monocyte chemoattractant protein-1 (MCP-1), and IL-6 which in turn stimulate switch of macrophages toward M2 phenotype, facilitating tumor growth [108]. Also, it has been observed that TGF-*β* produced by tumor cells [102] or soluble factors of M1 macrophages [108] can elevate production of iNOS by MSCs. In both cases, MSCs have demonstrated tumor-promoting and immunosuppressive activities. In contrary, it has been shown that iNOS-expressing MSCs can attenuate growth of fibrosarcoma cells [109]. According to this, it has been described that iNOS produced by stromal cells has dual role in cancer [110]. As role of M1 and M2 macrophages is important in early and late phase of the tumor development [47, 111], it is possible that similar trend can occur in MSCs. Although there is no evidence that MSCs can contribute to M1 polarization of macrophages, presence of iNOS-expressing M1 macrophages within TME makes speculating whether MSCs in TME can also achieve M1 phenotype possible. Therefore, iNOS can be considered as switch molecule of functional states of macrophages and MSCs in TME. However, increased expression of iNOS in MSCs must be carefully studied in context of tumor development. Taken together, these observations nicely exemplify very perplexed collaboration of MSCs and macrophages in TME [107].

Additionally, it has been found that mouse MSCs derived from lymphomas induce stronger recruitment of CD11b^+^Ly6C^+^ monocytes, F4/CD80^+^ macrophages, and CD11b^+^Ly6G^+^ neutrophils toward tumor tissue in comparison to healthy bone marrow MSCs. However, in this study it has been reported that lymphoma-derived MSCs promote tumor growth, expressing high levels of CCR2 ligands which lead to accumulation of monocytes and macrophages. Accumulation of monocytes and macrophages, but not neutrophils, has been essential for tumor progression. In presence of TNF-*α*, healthy bone marrow MSCs have been shown to possess similar effects on monocytes and macrophages recruitment as lymphoma-derived MSCs, thus contributing to tumor growth. These results suggest that lymphoma MSCs more potently support tumor growth than bone marrow MSC, while TNF-*α* could induce tumor-promoting effects of healthy bone marrow MSCs [112].

Reciprocal interactions of tumor-associated neutrophils (TANs) and MSCs in TME are not studied in detail, although it has been assumed that TANs can support or inhibit tumor development. It has been previously reported that cocultivation of mouse TNF-*α* treated bone marrow MSCs induced immunosuppressive functions of CD11b^+^Ly6G^+^ neutrophils. Cocultivation with MSCs increases arginase activity and expression of iNOS in neutrophils, promoting their capacity to inhibit proliferation of T lymphocytes and leading to stimulation of tumor growth* in vivo*. These results indicate that MSCs can promote immunosuppressive function of neutrophils which contribute to tumor development [113]. Moreover, it has been observed that cooperation of TANs and TAFs in follicular lymphoma contributes to promotion of malignancy of lymphoma cells. Namely, it has been found that TAFs presented in lymphoma produce high level of IL-8 which stimulated neutrophil survival. In turn, neutrophils activate TAFs through NF-*κ*B activation, leading to proinflammatory TAF phenotype generation, which increase survival of malignant B-cells, as well as recruitment of monocytes and neutrophils [114].

Immature cells of myeloid lineage, including monocytes, macrophages, neutrophils, and dendritic cells, participate in one heterogeneous population of MDSCs. Despite absence of harmonized panel of characterization markers for MDSCs, expansion of these cells has been detected in various tumor tissues [115]. MDSCs have been shown to inhibit proliferation of T lymphocytes and stimulate generation of Treg, thus contributing to immunosuppressive milieu of TME. Recently, it has been observed that pathological conditions of multiple myeloma can induce altered functional profile of MSCs. Namely, it has been described that human MSCs derived from healthy donors can induce generation of granulocytic-MDSCs, while only MSCs derived from multiple myeloma patients induce expansion of granulocytic-MDSCs with immunosuppressive capacity. Multiple myeloma MSCs have induced expression of high levels of arginase-1, TNF-*α*, and angiogenic factor PROK2 in MDSCs, thus contributing to tumor development [116]. Additionally, it has been demonstrated that human umbilical cord blood MSCs secret the chemokines growth-regulated oncogene CXCL1, CXCL2, and CXCL3 which affect differentiation and function of human monocyte-derived dendritic cells. In this study, it has been revealed that MSCs mitigate maturation of monocyte-derived dendritic cells driving their differentiation toward MDSCs* in vitro*. Namely, exposure of MDSCs to chemokines secreted by MSCs induces their immunosuppressive phenotype and production of increased levels of IL-10 and IL-4 and reduced levels of proinflammatory cytokines IL-12 and IFN-*γ*. In this study it has been demonstrated that mouse bone marrow MSCs stimulate differentiation of mouse bone marrow dendritic cells toward MDSC-like cells Gr-1^+^CD11b^+^ *in vivo*, enhancing their expression of the arginase-1 and iNOS mRNA, which contribute to their anti-inflammatory activity [117]. Furthermore, it has been observed that MSCs can stimulate expansion of CD14^−^CD11b^+^CD33^+^ MDSCs from peripheral blood leukocytes, by producing HGF that binds to c-Met on MDSCs, and consequently increase phosphorylation of STAT3, necessary for expansion of MDSCs [118]. In contrary, it has been observed that mouse bone marrow MSCs inhibited generation and proliferation of Gr-1^+^CD11b^+^ MDSCs in peripheral blood and bone marrow, and these effects have been suggested to be related to suppression of tumor growth* in vitro* and* in vivo* [119]. Thus, further investigations are necessary to elucidate interactions of MSCs and MDCSs as well as consequences of their interplay on tumor development.

### 10.2. Adaptive Immune Cells

Due to their ability to suppress proliferation of effector T lymphocytes, potential use of MSC in allogeneic transplantation experimental and clinical settings has been investigated for long time [120]. As has been noticed that coadministration of MSCs with hematopoietic stem/progenitor cells into patients with hematologic malignancies can cause relapsed leukemia or development of various tumors, potential use of MSCs in treatment of graft-versus-host disease is still questionable [121]. However, today it is clear that MSCs change not only proliferation, but also differentiation and maturation of T lymphocytes. It has been proposed that various soluble factors are involved in MSC-mediated immunosuppression such as indoleamine 2,3-dioxygenase (IDO), prostaglandin E2 (PGE2), iNOS, and TGF-*β* which can reduce proliferation of T lymphocytes. Also, it has been demonstrated that MSCs which have been stimulated to express high level of IDO-1, promote tumor growth* in vitro* and* in vivo*. Moreover, it has been shown that MSCs significantly impair infiltration of CD8^+^ T lymphocytes in tumor tissue. These results indicate that immunosuppressive role of MSCs in TME is at least partly mediated by IDO-1 [122]. Also, it has been reported that MSCs can provide immune protection to breast cancer cells. Namely, TGF-*β* produced by MSCs inhibits proliferation and functions of cytotoxic CD8 lymphocytes and CD56^+^ NK cells, while stimulating generation of Treg [123]. Interestingly, it has been observed that TGF-*β* produced by MSCs can induce generation of Treg from naïve T cells through Smad2 activation. These effects of MSCs contribute to prevention of colitis (inflammation stage) and colitis-associated colorectal cancer development (tumor stage) [124]. Also, MSCs can direct outcome of hematologic malignancies, altering repertoire of immune cells. In case of follicular lymphoma, it has been demonstrated that MSCs sustain viability of follicular helper T-cells, follicular regulatory T-cells and Treg, and generation of follicular regulatory T-cells from follicular helper T-cells, in IL-6-dependent manner [125].

Proliferation of malignant B cells in chronic lymphocytic leukemia (CLL), follicular lymphoma (FL), mucosa-associated lymphoid tissue (MALT) lymphomas, and multiple myeloma (MM) is mainly dependent on extrinsic stimuli from microenvironment, antigens, cytokines, and cell-cell interactions which are proposed to be responsible for nonefficacy of therapeutics [126]. It has been reported that stromal cells produce cytokines IL-4 and IL-21, which bind to interleukin receptor on lymphoma cells (IL-4R/IL-21R) or chemokines CXCL12 and CXCL13, thus forming the complex which is also implicated in evolution of tumor microenvironment and moreover in acquirement of malignant properties by cells [127]. There are reports emphasizing that interactions with bone marrow MSCs are critical for survival of malignant B cells, regulating apoptosis-regulatory protein Bcl-2 expression. However, MSCs have been shown as key factor of survival of normal as well as malignant B cells in CLL and acute lymphoblastic leukemia [128]. Also, it has been found that MSCs produce IL-6 which stimulates proliferation and differentiation of B cells in multiple myeloma [129]. In case of mantle cell lymphoma, it has been demonstrated that long term cocultivation with stromal cells contributes to drug-resistance of primary mantle cell lymphoma cells, partly via NF-*κ*B activation in B cells which lead to increased survival, migration, and drug resistance [130]. Since MSCs regulate proliferation and differentiation of hematopoietic stem and progenitor cells, it has been suggested that MSCs can also sense transformed HSCs and leukemic stem cells and regulate their expansion and disease development [131].

## 11. Antitumor Features of MSCs

Considering ability of MSCs to home to tumor tissue, their use as novel tool in antitumor therapy has been suggested. Namely, it has been shown that human umbilical cord MSCs, previously modified for high expression of IL-18 gene, inhibit proliferation and invasiveness of breast cancer cells. It has been proposed that MSCs expressing IL-18 inhibit proliferation of breast cancer cells by alteration of their cell cycle [132]. Additionally, it has been reported that umbilical cord MSCs with previously enhanced IL-15 gene expression significantly suppresses pancreatic tumor growth in mice. Moreover, in this study it has been demonstrated that MSCs, induced for IL-15 expression, stimulate accumulation of NK cells and CD8^+^ T lymphocytes, thus supporting antitumor immune response [133]. Also, it has been shown that IFN-*β*-producing MSCs from bone marrow [134], as well as adipose tissue [135], inhibit proliferation of hepatocellular carcinoma or breast cancer, respectively. Additionally, it has been reported that IFN-*β*-producing bone marrow MSCs attenuate proliferation of hepatocellular carcinoma cells, modifying their cell cycle, decreasing expression of cyclin D1 and phosphorylation of Rb via suppressed Akt and stimulated FOXO3a activity [134]. On the other side, it has been shown that IFN-*β*-producing adipose tissue MSCs exert cytotoxic effect on breast tumor cells mediated by STAT1 activation [135].

Additionally, it has been shown that TNF-related apoptosis inducing ligand (TRAIL) can selectively trigger apoptosis in tumor cells [136]. Similar antitumor activity of MSCs has been observed* in vivo* in mouse xenograft model of intraperitoneal human mesothelioma where MSCs overexpressing TRAIL attenuate inflammation in TME [137]. Also, antitumor activity of MSCs expressing TRAIL has been reported in xenograft models of sarcomas [138] and breast cancer [139]. Considering iNOS gene therapy for cancer treatment, it has been demonstrated that human MSCs expressing iNOS can inhibit/delay growth of fibrosarcoma in xenograft mouse model. It is suggested that delivered iNOS can generate NO or other cytotoxic intermediate molecules affecting viability of tumor cells [109]. These data indicate that MSCs modified for expression of tumor-inhibiting factors potentially may represent new approach in targeted antitumor therapy.

## 12. Concluding Remarks

Maintenance of chronic inflammation in TME requires engagement of important cellular compartments: tumor cells, immune cells, and MSCs. The mechanisms of the interplay between these cells are currently the subject of intensive experimental investigation and many questions are remained open. Chronic inflammation associated with hypoxia in TME provides generation of specific niche for MSCs, adjusting their phenotype and immune-related properties, thus bringing additional complexity to regulation of MSCs and tumor cells communication. Although many studies have reported supportive role of MSCs in tumor development, some investigations invest efforts to utilize tumor-homing capacity of MSCs to mitigate inflammation in TME or stimulate antitumor immune response.
